# Towards an understanding of psychedelic-induced neuroplasticity

**DOI:** 10.1038/s41386-022-01389-z

**Published:** 2022-09-19

**Authors:** Abigail E. Calder, Gregor Hasler

**Affiliations:** University Center for Psychiatric Research, University of Fribourg, Fribourg, Switzerland

**Keywords:** Long-term potentiation, Depression, Adult neurogenesis, Neurotrophic factors

## Abstract

Classic psychedelics, such as LSD, psilocybin, and the DMT-containing beverage ayahuasca, show some potential to treat depression, anxiety, and addiction. Importantly, clinical improvements can last for months or years after treatment. It has been theorized that these long-term improvements arise because psychedelics rapidly and lastingly stimulate neuroplasticity. The focus of this review is on answering specific questions about the effects of psychedelics on neuroplasticity. Firstly, we review the evidence that psychedelics promote neuroplasticity and examine the cellular and molecular mechanisms behind the effects of different psychedelics on different aspects of neuroplasticity, including dendritogenesis, synaptogenesis, neurogenesis, and expression of plasticity-related genes (e.g., brain-derived neurotrophic factor and immediate early genes). We then examine where in the brain psychedelics promote neuroplasticity, particularly discussing the prefrontal cortex and hippocampus. We also examine what doses are required to produce this effect (e.g., hallucinogenic doses vs. “microdoses”), and how long purported changes in neuroplasticity last. Finally, we discuss the likely consequences of psychedelics’ effects on neuroplasticity for both patients and healthy people, and we identify important research questions that would further scientific understanding of psychedelics’ effects on neuroplasticity and its potential clinical applications.

## Introduction

Recent decades have seen renewed scientific interest in classic psychedelics, which include lysergic acid diethylamide (LSD), psilocybin, 2,5-dimethoxy-4-iodoamphetamine (DOI), 5-methoxy-N,N-dimethyltryptamine (5-MeO-DMT), and N,N-dimethyltryptamine (DMT), the psychedelic compound in the Amazonian ayahuasca brew [1]. Classic psychedelics have been shown to catalyze relatively long-lasting improvements in mental health after a small number of doses, especially when combined with psychotherapy [2]. In patients suffering from depression, anxiety disorders, and addiction, the benefits of psychedelic-assisted psychotherapy can last for many months or years [3–10]. Additionally, healthy subjects report increased well-being up to a year after administration of psychedelics in a safe and supportive setting [11–13].

One leading theory of psychedelics’ lasting effects categorizes them as “psychoplastogens” which rapidly stimulate a period of enhanced neuroplasticity, as well as enduring neuroplastic changes [14, 15]. Neuroplasticity denotes the nervous system’s ability to reorganize its structure and function and adapt to its dynamic environment [16]. Throughout the lifespan, neuroplasticity is essential for learning, memory, and recovery from neurological insults, as well as adapting to life experiences [17]. The theory that psychedelics open a window of neuroplasticity would explain how long-term effects outlast the drug’s presence in the body, and it is also attractive because disruptions in neuroplasticity are present in mood disorders and addiction [18].

Neuroplasticity can be investigated at multiple levels of analysis. At the molecular level, it comprises changes in gene and protein expression, as well as post-translational modifications [19]. Of particular importance is brain-derived neurotrophic factor (BDNF), a neurotrophin that regulates neuronal growth and synaptic plasticity [20]. Changes in gene and protein expression give rise to morphological changes, including the formation and modification of synapses and dendrites [21]. In particular regions, most notably the hippocampus, neuroplasticity also comprises neurogenesis [22]. These processes modify neural circuits, ultimately manifesting in learning, memory, and changes in adaptive behavior [19]. Neuroplasticity is crucially activity-dependent at the cellular level, which translates into experience-dependence at the level of cognition and behavior: people learn both passively and actively from their experiences, adjusting patterns of thought, emotion, and behavior accordingly [17, 23].

In order to effectively harness the potential of psychedelics, it is imperative to understand how they affect neuroplasticity, as well as the clinical relevance of these effects. In the present review, we first evaluate the available evidence concerning whether psychedelics enhance neuroplasticity. We then discuss where in the brain this likely happens, what doses are capable of this, how long the effects may last, and whether they have meaningful consequences for emotion, cognition, and behavior, as well as therapeutic use. Finally, we discuss the advantages and challenges that psychedelic-induced neuroplasticity presents and identify important directions for future research.

## Do psychedelics enhance neuroplasticity?

Classic psychedelics are thought to catalyze a period of accelerated neuronal growth, enhancing the brain’s capacity for neuroplastic changes. Studies in animals have shown that LSD, psilocybin, DMT, and DOI promote the expression of genes related to synaptic plasticity, including immediate early genes (IEGs) and *BDNF* [24–34]. Furthermore, they catalyze a burst of synaptic and dendritic growth and can increase the strength of long-term potentiation (LTP) [27, 35–40]. Regarding neurogenesis, results have been mixed: LSD and DOI had no effect on adult neurogenesis in rats, and psilocybin was shown to slightly reduce it in mice [41–43]. By contrast, studies in mice using both DMT and 5-MeO-DMT observed increased neurogenesis [44, 45].

In humans, studies have often relied on peripheral BDNF as a marker of neuroplasticity, yielding mixed results. Though ayahuasca increased BDNF levels in both healthy and depressed people in one study, another found no change [46, 47]. Several studies have measured the effects of LSD on BDNF, with some finding an increase [48, 49] and others no change [50, 51]. In two studies in healthy subjects, comparable doses of psilocybin did not elevate plasma BDNF in one [50], but did so in the other [52]. This variability may be partially due to the limitations of peripheral BDNF as a biomarker in pharmacological studies. Though blood BDNF has been shown to predict brain BDNF under normal conditions, psychoplastogens may cause an increase in peripheral BDNF levels without any increase in brain BDNF [53, 54]. Furthermore, BDNF may not correlate with other measures of cortical neuroplasticity in humans, and blood platelets can store and release BDNF independently of neurons [55, 56]. Beyond measuring BDNF levels, neuroimaging studies have found evidence of altered neural connectivity following treatment with psilocybin and ayahuasca, which is interpreted as evidence of drug-induced neuroplastic changes [57–59].

Taken together, animal studies offer moderately strong evidence that psychedelics promote genes related to neuroplasticity, synaptic strength, and dendritic growth, including *BDNF*. However, analyses of peripheral BDNF protein in human studies have thus far been inconclusive. Future studies in humans could benefit from protocols which do not rely only on peripheral markers, but also induce LTP-like changes to index neuroplasticity, such as paired associative stimulation [60–62] or tetanic sensory stimulation [63, 64], as well as PET studies with markers of synaptic density, such as SV2A [65].

## How do psychedelics enhance neuroplasticity?

The complex molecular signaling underlying psychedelic-enhanced neuroplasticity has been thoroughly discussed elsewhere [66–69], but we will briefly review the most important aspects. Psychedelics appear to enhance neuroplasticity via the 5- HT_2A_ receptor, which also mediates most of their subjective effects [70–72]. Though relatively low doses of the selective 5-HT_2A_ receptor antagonist ketanserin do not fully block psychedelic-induced neuroplasticity [37, 73], higher doses of ketanserin block it completely [36]. Furthermore, the affinity of different psychedelic drugs for the 5-HT_2A_ receptor predicts their individual potency as psychoplastogens, and 5-HT_2A_ receptor knockout mice show no signs of enhanced neuroplasticity following treatment with psychedelics [24, 27, 36].

Psychedelics stimulate 5-HT_2A_ receptors found post-synaptically on layer 5 and 6 pyramidal neurons, as well as on GABAergic interneurons [72]. The net effect appears to be excitation of layer 5 pyramidal neurons and increased levels of extracellular glutamate, resulting in greater stimulation of AMPA receptors [35, 72, 74]. The precise molecular pathways which may modify neuroplasticity after 5-HT_2A_ receptor stimulation are not fully understood. However, one leading hypothesis suggests that the aforementioned AMPA receptor stimulation triggers a positive feedback loop: Stimulation of AMPA receptors may enhance BDNF secretion, which would stimulate TrkB receptors and mTOR, which in turn would stimulate further BDNF production and sustained AMPA activation [36, 38]. Sustained activation of both AMPA receptors and mTOR appears to be necessary for the enhanced dendritic growth following stimulation with psychedelics [35]. Additionally, activity involving both 5-HT_2A_ and glutamate receptors, particularly mGlu_2_, may be essential for psychedelics’ effects on neuroplasticity [66, 75, 76]. These effects likely remain specific to synapses and circuits expressing 5-HT_2A_ receptors, as BDNF acts locally and does not diffuse far after release [20, 77].

In addition to 5-HT_2A_ receptors, the effects on neurogenesis seen with DMT and 5-MeO-DMT could potentially involve other receptors [42, 43]. DMT has low but functionally significant affinity for the sigma-1 receptor, an orphan receptor involved in neuroprotection and neurogenesis [78]. Sigma-1 receptor antagonists block DMT’s effects on hippocampal neurogenesis [44, 79], and sigma-1 receptor activity has also been shown to stimulate neurogenesis in previous studies [80–82]. The affinity of DMT for sigma-1 receptors may also not only its effects on neurogenesis, but also DMT’s neuroprotective effects in a rat model of stroke [83].

Concerning 5-MeO-DMT, this molecule is unusual among psychedelics in that it has a nearly 1000-fold higher affinity for 5-HT_1A_ than 5-HT_2A_ receptors, and many of its effects are mediated by 5-HT_1A_ receptors [79, 84–87]. Hippocampal 5-HT_1A_ receptors may drive neurogenesis, suggesting that the effects of 5-MeO-DMT on neurogenesis could conceivably occur via potent, relatively selective activation of 5-HT_1A_ receptors [88, 89]. Additionally, 5-HT_1A_ receptors are generally inhibitory and tend to have opposite effects on downstream signaling pathways than 5-HT_2A_ receptors [90–93]. Many psychedelics show binding affinity for both 5-HT_2A_ and 5-HT_1A_ receptors [94]. Furthermore, some of psychedelics’ effects on attention and the visual system may be mediated by the 5-HT_1A_ receptor [95, 96]. The excitatory and neuroplastic effects of different psychedelic drugs in any particular brain region could conceivably depend on whether that region is richer in 5-HT_2A_ or 5-HT_1A_ receptors [79, 97–101].

## Where do psychedelics enhance neuroplasticity?

Because psychedelics promote synapse and dendrite growth in a 5-HT_2A_ receptor-dependent manner, the greatest effects would be expected in regions with high 5-HT_2A_ receptor expression, i.e., the neocortex [72, 91, 102]. Data from animal studies thus far supports this theory, showing relatively robust effects in cortical regions and smaller, less consistent effects on neuroplasticity elsewhere.

### Neocortex

Psychedelics have been shown to enhance dendritic growth, including spinogenesis, in cortical neurons [36, 40]. In the frontal lobe specifically, animal studies show that psychedelics upregulate plasticity-related genes and promote the growth of synapses and dendritic spines [25, 27, 36, 37, 103]. In the prefrontal cortex (PFC), several psychedelics have been shown to rapidly upregulate genes related to neuroplasticity [25, 26, 104]. Pigs exposed to a hallucinogenic dose of psilocybin showed increased presynaptic density in the PFC [39]. In humans, PET imaging has shown that psilocybin increases glutamate signaling in the PFC, which is theorized to be important for psychedelic-enhanced plasticity [105].

Other cortical regions likely also show enhanced neuroplasticity as a function of 5-HT_2A_ receptor density. DOI enhanced expression of the plasticity-related *Arc* gene in the whole cortex, as well as in the parietal cortex specifically [28, 106]. A recent unpublished study in mice examined expression of *c-Fos*, an early marker of neuroplastic processes, after treatment with psilocybin, revealing strong upregulation in most cortical regions. These included sensory visual, auditory, somatosensory, and gustatory areas, as well as motor and association areas, the anterior cingulate cortex (ACC), and the insula [107].

### Hippocampus

Several studies have focused on the hippocampus, but many found modest effects compared to the cortex. In the rodent hippocampus, psilocybin treatment upregulated fewer plasticity-related transcripts in the hippocampus than in the cortex, and LSD failed to upregulate immediate early genes associated with neuroplasticity [24, 25]. Similarly, DOI failed to enhance expression of *Arc* in the hippocampus [106]. Treatment with DOI may even decrease the expression of *BDNF* in the dentate gyrus, leaving it unchanged in the rest of the hippocampus [28]. In line with this, the abovementioned PET study in humans found reduced glutamate activity in the hippocampus after psilocybin [105]. However, the cortex and hippocampus do not always show this opposite pattern. Pigs exposed to a hallucinogenic dose of psilocybin showed increased presynaptic density in both the hippocampus and the PFC [39]. Additionally, psilocybin has been shown to strengthen cortico-hippocampal synapses [73].

The reduced tendency toward neuroplastic effects in the hippocampus might be explained by its greater density of 5-HT_1A_ than 5-HT_2A_ receptors [90, 102]. It is possible that LSD, DOI, and psilocybin, and perhaps other psychedelics, have pro-neuroplastic effects in the cortex and other regions richer in 5-HT_2A_ than 5-HT_1A_ receptors, but tend to have modest or even inhibitory effects in 5-HT_1A_ receptor-dominant areas like the hippocampus.

### Other subcortical regions

Some preliminary unpublished evidence suggests that psychedelics may enhance neuroplasticity in a few subcortical regions. In the aforementioned study of *c-fos*, psilocybin increased *c-fos* expression in the claustrum, locus ceruleus, lateral habenula and some areas of the thalamus, amygdala, and brainstem [107]. The pattern of expression changes correlated with 5-HT_2A_ receptor distribution [107]. Given that *c-fos* is a relatively unspecific marker, however, these results should be interpreted with caution, and more research is necessary to determine how psychedelics affect neuroplasticity in subcortical regions.

The mesolimbic pathway warrants particular attention due to its role in addiction. Addiction to drugs of abuse is driven by neuroplastic changes in dopaminergic neurons of the mesolimbic pathway [108]. Notably, however, psychedelics do not cause dependence or addiction [108]. Important mesolimbic areas for addiction, including the ventral tegmental area, nucleus accumbens, and striatum, express relatively few 5-HT_2A_ receptors and are therefore unlikely to be much affected by psychedelic-induced plasticity [102, 109]. Additionally, inhibitory neurons projecting from the PFC to areas of the mesolimbic pathway are much richer in 5-HT_2A_ receptors [102, 110], and enhanced dendritic growth in these PFC neurons could conceivably contribute to the anti-addictive effect observed with psychedelics [3, 10, 111].

### At what dose do psychedelics enhance neuroplasticity?

Several studies have investigated how different doses of psychedelic drugs affect neuroplasticity. In rats, 0.2 mg/kg LSD promoted neuroplasticity-related changes in gene expression, and the efficacy increased up to a dose of 1 mg/kg, although some genes showed a peak effect at lower doses [31–33]. For psilocybin, a dose of 4 mg/kg was required to induce neuroplasticity-related changes in gene expression, and the effect also increased in a dose-dependent manner [25]. DOI also shows a dose-dependent effect on neuroplasticity [28]. Finally, a presumably sub-hallucinogenic dose of 1 mg/kg DMT increased functional plasticity in rat cortical slices, as measured by the frequency and amplitude of excitatory post-synaptic currents [36].

Though these studies suggest that psychedelics probably promote neuroplasticity in a dose-dependent manner, clear dose-response effects on neuroplasticity have not been established in humans. Sub-hallucinogenic doses of between 5 and 20 µg LSD produced significant short-term enhancements in plasma BDNF [48]. However, a similar study using doses of between 25 µg and 200 µg LSD only found significant effects on BDNF at 200 µg [49], and another failed to find significant changes even at this dose [50]. Perhaps using different methods, future research should seek to clarify the minimum and optimal doses for stimulating neuroplasticity with different psychedelics. The prospect of non-hallucinogenic “microdoses” which enhance neuroplasticity is attractive for certain clinical applications, including stroke, brain injury, and neurodegenerative disorders [15].

Particularly regarding microdoses, a discussion of dosing frequency is warranted. While large doses of psychedelics are not taken chronically due to their intense subjective effects, microdoses can be taken regularly and have been hypothesized to enhance neuroplasticity [48, 112]. Chronic dosing with LSD has been associated with enhanced eyeblink conditioning, as well as improved avoidance learning and reversal of stress-induced deficits in synaptogenesis in rodent models of depression [103, 113, 114]. However, chronic dosing with DMT may cause retraction of dendritic spines [115]. Additionally, chronic LSD dosing was associated with upregulation in genes related to neuroplasticity, but also to schizophrenia [104]. Many animal studies investigating chronic dosing have not differentiated between microdoses and hallucinogenic doses, which may be an important distinction. Nevertheless, further studies should investigate whether chronic dosing, particularly chronic microdosing, has different effects on neuroplasticity than single doses.

### For how long do psychedelics enhance neuroplasticity?

**Fig. 1:**
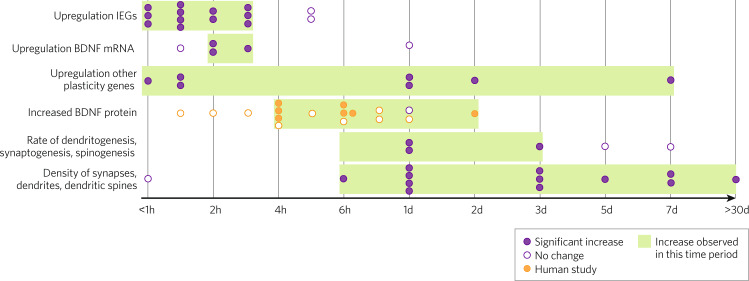
Timeline showing the earliest and latest observations of various changes in neuroplasticity following treatment with a single dose of the serotonergic psychedelics LSD, psilocybin/psilocin, DMT, or DOI. One dot represents one study and time point. Human studies are shown in yellow; animal and in vitro studies are shown in purple. BDNF = brain-derived neurotrophic factor, IEGs = immediate early genes. Based on data for synaptic density, it is assumed that rates of dendritogenesis and synaptogenesis also increase at 6 h post-treatment. See Table S1 for citations.

In order to take advantage of a “window of plasticity,” it is essential to know when this window opens and closes. Evidence of enhanced neuroplasticity appears within several hours after exposure to psychedelics (Fig. 1). The earliest changes involve upregulation of neuroplasticity-related transcripts, which can occur within one hour [24, 34]. In rats, both LSD and psilocybin upregulated genes associated with neuroplasticity after 1.5 hours, particularly in the PFC [25, 33]. *BDNF* mRNA may become upregulated slightly later: one study found no change 1.5 hours after treatment with psilocybin, but others have found increased expression 2 and 3 hours after treatment with DOI [25, 28, 116].

Changes in cellular morphology have been observed starting 6 hours after stimulation with psychedelics [35]. One study found no changes in dendritic growth 1 hour after stimulating primary rat neuronal cultures with LSD, but observed significant changes in dendritic growth, synaptogenesis, and spinogenesis at several later time points [35]. In humans, increases in peripheral BDNF levels have earliest been seen 4 hours after oral administration of LSD [48, 49].

Though neuroplasticity may increase within several hours, the peak effect may come some time later. In rat cortical neurons, the observed increase in synaptogenesis was greater at 24 hours than at 6 hours post-stimulation, and in female mice, the rate of dendritic spine formation 3 days after psilocybin treatment is greater than the rate seen just 1 day after treatment [36, 37]. Other work has shown that a significant neuronal growth phase occurs in the 72 hours after initial exposure to psychedelics [35].

Enhanced neuroplasticity may also last for several days. In mice treated with psilocybin, the rate of dendritic spine formation remained elevated for 3 days, returning to baseline by 5 days post-treatment [37]. In humans, both healthy volunteers and depressed patients show elevated peripheral BDNF levels 2 days following treatment with ayahuasca [46]. Finally, a study that treated mice with LSD every other day for 1 month observed long-term upregulated of neuroplasticity-related genes, including *BDNF*, in the medial PFC 4 weeks after treatment cessation [104]. Additionally, specific markers of neuroplasticity may have different “windows.” Though *BDNF* mRNA can become upregulated within 2 hours, the effect may already be gone 24 hours later, and it is unclear what this means for BDNF protein expression [36]. Upregulation of other plasticity-related genes follows various time courses, with some genes showing peak expression within a few hours, others at around 48 hours, and still others at 7 days after administration [27, 31, 32].

Crucially, new dendrites and synapses formed during the window of enhanced neuroplasticity can outlast the window itself. Increased synaptic and dendritic density has been observed at 72 hours post-treatment in multiple studies [27, 37, 39]. Furthermore, though mice treated with psilocybin returned to baseline levels of dendritic spine formation within 5 days, new dendrites formed during that period survived for at least 1 month [37]. In humans, research has uncovered changes in brain function which lasted at least 1 month after treatment with psilocybin, suggesting the presence of lasting neuroplastic changes [57].

These data suggest that various signs of enhanced neuroplasticity arise within 1–6 hours, with changes in gene expression appearing earliest and changes in cell morphology and synapse organization arising later. The increased rate of dendritogenesis may taper off within 5 days, however, neuroplastic changes which arise during this period of neural growth may last for at least 1 month. However, important questions about the window of neuroplasticity remain, and future research should aim to define the temporal dynamics of enhanced neuroplasticity in humans, as this may be crucial for the timing of psychotherapeutic interventions.

### Consequences of enhanced neuroplasticity

Is enhanced neuroplasticity simply something we can measure, or does it also have meaningful consequences? Answering this question is essential for understanding the basis of psychedelics’ long-term effects, however, few studies have related changes in neuroplasticity directly to behavioral outcomes. In chronically stressed mice, psilocybin both strengthened cortico-hippocampal synapses and reduced anhedonia, which may be the result of improved synaptic strength in reward circuits [73]. Additionally, DMT has been seen to enhance both neurogenesis and memory performance [44]. Other studies have reported improvements in fear extinction learning and reductions in anxious behaviors and learned helplessness following exposure to psychedelics, while also observing increased dendritic spine density in separate cohorts of animals [27, 37, 103]. Finally, the enhanced spinogenesis induced by ketamine, which is also a psychoplastogen, has been associated with reductions in depression-related behaviors [117, 118]. More research is needed to determine whether the same could be true for classic psychedelics, and to confirm or deny the associations between neuroplastic and behavioral effects suggested in the literature thus far.

In humans, one study found that depressed patients treated with ayahuasca had elevated BDNF levels which correlated with their clinical improvements [46]. In another study, psilocybin lastingly increased connectivity between the PFC and other brain areas, including limbic and subcortical regions, and these increases occurred alongside decreases in negative affect and anxiety [57]. However, one limitation of many of these studies is the lack of causal inference: though changes in neuroplasticity and changes in cognition or behavior may occur simultaneously, whether neuroplasticity mediated those changes remains an open question for future studies to address.

### Further outcomes possibly explained by enhanced neuroplasticity

Changes in neuroplasticity may also partially explain some other long-term effects of psychedelics. Psychedelics, combined with psychotherapy, have shown clinical efficacy in trials for mood disorders and addiction, and healthy participants also report improved mood after taking psychedelics [3–6, 10, 119–123]. Enhanced dendritic and synaptic growth in PFC neurons may be a plausible explanation for this: the PFC is essential for emotional regulation via its connections with the amygdala and other subcortical regions [124, 125]. Depression in particular is characterized by reduced cortical neuroplasticity [56, 126–128], synapse atrophy in the PFC [18, 129–131], and a reduced ability of the PFC to regulate limbic areas [132, 133]. Additionally, PTSD, social anxiety disorder, and generalized anxiety have been associated with fewer synaptic connections between the medial PFC and the amygdala, compromising the PFC’s ability to regulate fear responses [134–136]. In addiction, neuroplasticity in the circuits between the PFC and the nucleus accumbens, striatum, and limbic system becomes impaired, reducing PFC modulation of these regions [137]. Relatively selective dendritic growth on neurons originating in the PFC may help reverse these deficits, restoring signaling balance and top-down control over the limbic system.

Other modest cognitive improvements found after treatment with psychedelics may also be explained by enhanced neuroplasticity in cortical regions. In animal studies, chronic LSD treatment has been associated with improvements in learning [113, 114, 138]. In humans, LSD has improved frontal-dependent memory retrieval, and unpublished data suggests that it may also improve reinforcement learning, possibly by enhancing reward sensitivity [139, 140]. Cognitive flexibility also involves several circuits originating in the PFC [141, 142], and ayahuasca and psilocybin have been shown to promote certain aspects of cognitive flexibility [143–147]. Regular ayahuasca users additionally perform better on tests of behavioral inhibition, cognitive flexibility, working memory, and executive functioning [147]. Ayahuasca and psilocybin have also been shown to increase mindfulness, one form of attentional regulation for which the PFC, but also the ACC is essential [13, 58, 143, 148–150]. It is possible that dendritic growth in PFC and ACC neurons is responsible for these effects [59].

Finally, neuroplasticity may not only play a role in positive long-term effects of psychedelics, but also undesirable ones. Drug-induced neuroplastic changes in sensory regions could conceivably be a factor in psychedelic-induced flashbacks, as well as the rarer and more severe hallucinogen persisting perceptual disorder (HPPD), in which some drug effects, including hallucinations and psychological distress, persist after the drug has been metabolized [108, 151].

### Experience-dependent neuroplasticity

Neuroplastic changes occur in an activity- and experience-dependent manner [16]. This is an important consideration when discussing psychedelic-enhanced neuroplasticity, because psychedelics themselves can catalyze intense experiences [2]. The beginning of the window of plasticity falls within the timeline of many psychedelic drugs’ subjective effects, meaning that at least some of the psychedelic experience takes place within a highly plastic brain [1, 152].

Because of this, the experiences people have under psychedelics may have more power to re-shape neural circuitry than everyday occurrences. This possibility comes with opportunities and challenges. In a safe and supportive setting, psychedelic drugs can cause personally meaningful, emotionally salient experiences which can lead to lasting improvements in well-being [11]. Both patients and healthy volunteers report insights into personal problems, emotional breakthroughs, reprocessing of traumatic memories, and feelings of connectedness and empathy for oneself and others [7, 12, 123, 153–156]. Sometimes this can take the form of a “helioscope effect” in which people seem to perceive their experiences in more detail, but are also able to work through difficult material without becoming overwhelmed [157]. These effects are commonly described in terms of learning experiences [154, 158]. Furthermore, mystical experiences, emotional breakthroughs, and insights correlate significantly with positive long-term effects, independently from the overall intensity of drug effects [155, 159]. There may be a synergy between enhanced neuroplasticity and these positive, therapeutic experiences.

However, especially in unsafe settings, psychedelics can also cause “bad trips” involving intense physical and psychological distress [160]. Negative psychedelic experiences, in particular longer ones, are sometimes associated with subsequent negative changes in well-being, and feelings of anxiety during a psychedelic experience correlate negatively with therapeutic effects [12, 160–162]. Along these lines, most people who develop HPPD report that distressing symptoms appeared after a frightening acute psychedelic experience [163]. Crucially, not all negative experiences lead to decreases in well-being; in fact, most do not, and long-term negative effects are rare [12, 161]. In a survey of people who had had a challenging experience while on psilocybin, the duration of the challenging experience was significantly and negatively correlated with changes in well-being [161]. This suggests that challenging experiences which resolve relatively quickly are less likely to cause undesirable neuroplastic changes, perhaps because overcoming difficult feelings becomes a positive learning experience. However, prolonged experiences of anxiety and distress during a state of heightened plasticity have the potential to be damaging.

Finally, the psychedelic experience itself is not the only important experience in psychedelic therapy. Enhanced neuroplasticity may also make people more responsive to other therapeutic interventions, including psychotherapy, but potentially also neurorehabilitation after stroke or brain injury [14]. Therapeutic interventions combined with antidepressants, which also modestly promote neuroplasticity, have been shown to be more effective than either intervention alone, and the same is likely true of psychedelics [164, 165]. Enhanced neuroplasticity, coupled with a psychedelic experience in a safe setting and accompanying psychotherapy, could ultimately generate a therapeutic effect that is more than the sum of its parts.

## Conclusions

Significant progress has been made toward understanding how psychedelics affect neuroplasticity. Data thus far supports the theory that psychedelics stimulate dendritogenesis, synaptogenesis, and the upregulation of plasticity-related genes in a 5-HT_2A_ receptor-dependent manner, affecting the cortex in particular. The window of neuroplasticity appears to open within a few hours and may last a few days, although neuroplastic changes occurring during this time may survive for at least a month. Because neuroplastic changes occur in an experience-dependent manner, experiences people have during this time may have a greater psychological impact than they otherwise would. Future research should attempt to confirm preclinical findings in humans, clarify optimal doses and specific neuroplastic effects for different psychedelic compounds, and further explore the consequences of psychedelic-enhanced neuroplasticity for both patient groups and healthy people.

## Supplementary information

Table S1
